# Echocardiographic follow-up of patients with systemic sclerosis by 2D speckle tracking echocardiography of the left ventricle

**DOI:** 10.1186/1476-7120-12-13

**Published:** 2014-03-29

**Authors:** Sebastian Spethmann, Karl Rieper, Gabriela Riemekasten, Adrian C Borges, Sebastian Schattke, Gerd-Ruediger Burmester, Bernd Hewing, Gert Baumann, Henryk Dreger, Fabian Knebel

**Affiliations:** 1 Medizinische Klinik für Kardiologie und Angiologie, Campus Mitte, Charité Universitätsmedizin Berlin, Charitéplatz 1, Berlin D-10117, Germany; 2 Bundeswehrkrankenhaus Berlin, Abteilung I – Innere Medizin, Scharnhorststr. 13, 10115 Berlin, Germany; 3 Medizinische Klinik mit Schwerpunkt Rheumatologie und Klinische Immunologie, Campus Mitte, Charité – Universitätsmedizin Berlin, Charitéplatz 1, 10117 Berlin, Germany; 4 German Rheumatism Research Centre, a Leibniz Institute, Berlin, Germany; 5 Klinik für Innere Medizin I – Kardiologie, Helios Klinikum, Walterhöferstraße 11, 14165 Berlin, Germany

**Keywords:** Systemic sclerosis, Speckle tracking, 2D strain, Myocardial involvement

## Abstract

**Background:**

Subclinical myocardial involvement is common in systemic sclerosis (SSc) and associated with poor prognosis. Early detection, particularly during follow-up, is important. Two-dimensional speckle tracking echocardiography (STE) has already been shown to detect early left ventricular systolic impairment in SSc patients with advanced disease. The aim of this study was to assess the ability of STE to diagnose changes in left ventricular function in patients with SSc with preserved LV ejection fraction (LVEF) and normal pulmonary pressure over time.

**Methods:**

This single-center pilot study included nineteen SSc patients without pulmonary hypertension and preserved LVEF (55.2 ± 10.8 years, 13 women, mean modified Rodnan Skin Score of 8.2 ± 6.5, median disease duration 6 ± 4.5 years). We performed STE at baseline and after two years (mean 756.6 ± 8.8 days). Pulmonary hypertension was ruled out in all patients by right heart catheterization (average mean PAP 17.7 ± 3.5 mmHg).

**Results:**

The LVEF remained unchanged (63.3 ± 4.2% vs. 63.2 ± 5.0%, *P* = ns), but the global longitudinal peak systolic strain of the left ventricle was significantly lower: baseline -22.0 ± 2.3% vs. follow-up -20.8 ± 2.1% (*P* = 0.04). The regional analysis showed a heterogeneous distribution of segmental systolic dysfunction that did not match any particular coronary artery distribution. In contrast, the LV diastolic function remained stable during follow-up.

**Conclusion:**

STE might be a sensititive and valuable method to detect early LV systolic impairment in patients with SSc and preserved LVEF during two years. Prospective evaluations are needed for prognostic implications of these changes.

## Background

Clinical myocardial dysfunction in patients with systemic sclerosis (SSc), an entity characterized by extensive fibrosis, is recognized only in 15-25% [1,2]. In histological studies, however, myocardial involvement is more common [3,4]. In the myocardium, fibrosis tends to be patchy but distributed throughout the myocardium in both ventricles [1]. Importantly, cardiac involvement is one of the leading causes of disease-related death [2,5]. Fortunately, significant advances in symptomatic organ-specific therapy have been made during recent years [6]. Consequently, preclinical identification of myocardial manifestation is highly encouraged. At present, there is a lack of longitudinal evaluations concerning the presence and development of functional myocardial abnormalities. Echocardiography with newer modalitites like 2D speckle tracking (STE) allow assessment of myocardial deformation as a sensitive marker for regional and global LV systolic function and have already been analysed in SSc patients [7,8]. STE is a semi-automatic algorithm that can be carried out quickly in daily practise and is only minimally affected by inter- and intraobserver variability [9]. Since STE uses only standard 2D images, analyses can be performed offline from even already recorded examinations. These advantages let STE appear as an attractive method particularly for serial echocardiographic examinations.

The aim of this study was to assess subclinical changes in LV function by STE in SSc patients with preserved left ventricular ejection fraction (LVEF) and without pulmonary hypertension over a duration of two years.

## Methods

### Study population

21 SSc patients without pulmonary hypertension, reduced LVEF, or known coronary heart disease who were included in the DETECT (Detection of PAH in SSc) study [10] in our centre were screened. The study protocol was approved by the ethics committee of the Charité University Hospital. All participants provided written consent. One patient refused follow-up echocardiography and one had to be excluded due to mitral valve sclerosis at baseline. Therefore, nineteen patients with SSc according to the American College of Rheumatology classification criteria [11] (13 women, mean age 55.2 ± 10.8 years, range 33 to 74 years) with a median disease duration 6 ± 4.5 years (range 2 to 17 years) and a mean modified Rodnan Skin Score of 8.2 ± 6.5 were included into the study (Table 1). All patients underwent echocardiography with STE at baseline and 756.6 ± 8.8 days later to analyse global and regional systolic LV function. Pulmonary hypertension was ruled out in all patients by right heart catheterization [12] (average mean PAP 17.7 ± 3.5 mmHg). Table 1 shows the baseline characteristics in detail.

**Table 1:** Clinical and biological characteristics of SSc patients

	**Systemic sclerosis (n = 19)**
Age, (years)	55.2 ± 10.8
Sex (female), [n (%)]	13 (68.4%)
Disease duration, (years)	60 ± 4.5
Time of follow-up examination after baseline examination, (days)	756.6 ± 8.8
Time of baseline echocardiography between right heart catheter, (days)	6.0 ± 10.4
Body mass index (kg/m^2^)	24.0 ± 2.7
Arterial hypertension, [n (%)]	4 (21.1)
Smoker, [n (%)]	2 (10.5)
Raynaud’s syndrom, [n (%)]	8 (42.1)
Modified Rodnan Skin Score	8.2 ± 6.5
Anti-centromere antibodies, [n (%)]	4 (21.1)
Anti-Scl-70 antibodies, [n (%)]	7 (36.8)
Pulmonary fibrosis, [n (%)]	8 (42.1)
Impaired renal function, [n (%)]	2 (10.5)
***Invasive measurements***	
Cardiac output, (l/min)	5.4 ± 0.7
Cardiac index, (l/min/m^2^)	3.0 ± 0.4
PAP systolic, (mmHg)	27.3 ± 4.9
PAP diastolic, (mmHg)	11.2 ± 3.1
PAP mean, (mmHg)	17.7 ± 3.5
PCWP mean, (mmHg)	10.1 ± 3.6
SVR, (dyn × sec × cm^-5^)	2203.3 ± 787.7
PVR, (dyn × sec × cm^-5^)	188.3 ± 98.6

Data are expressed as mean ± SD, except gender, hypertension, and except disease duration and time of follow-up examination, which are expressed as median ± SD. SSc, systemic sclerosis; PAP, pulmonary artery pressure; PCWP, pulmonary capillary wedge pressure; SVR, systemic vascular resistance; PVR, pulmonary vascular resistance.

### Echocardiography and conventional Doppler measurements

Standard transthoracic echocardiography was performed in the left decubitus position using an ultrasound system (Vivid 7, GE Medical Systems, Horton, Norway) with a 3.4-MHz multifrequency transducer. The LVEF was obtained according to the recommendations of the ASE [13].

Left ventricular diastolic function was assessed using pulsed-wave Doppler and pulsed-wave Doppler tissue imaging (DTI) recordings on the basis of the recommendations of the ASE. Transmitral flow was acquired to obtain peak early (E) and atrial (A) flow velocities. We used the average peak early diastolic (E’), peak late diastolic (A’) and peak systolic velocity (S’) obtained from the septal and lateral sides of the mitral annulus in the fourchamber view with proper DTI settings. The E/E’ ratio was calculated to estimate LV filling pressures.

### 2D speckle tracking strain analysis

For assessment of longitudinal strain, we recorded standard 2D ultrasound images with a frame rate between 60 and 80 frames per second (fps) from the apical long axis, and two- and four-chamber views. We stored these recordings digitally for offline analysis (EchoPac PC, Version 112.1.1, GE Vingmed, Horton, Norway) as previously described [14]. In short, we used a semi-automatic algorithm for tracking the left ventricular myocardial wall, which was divided into 18 segments to obtain the global peak systolic longitudinal strain.

### Inter- and intraobserver variability analysis

Two echocardiographers, blinded to previously obtained data, separately measured global PSS from 13 random patients for interobserver variability analysis. Additionally, an experienced observer calculated strain values twice on two consecutive days for analysis of intraobserver variability. We employed inter- and intraobserver variability to determine the interclass coefficient.

### Statistics and figures

All results are expressed as mean ± standard deviation (SD). Statistics were calculated using SPSS 21.0 (IBM Corporation, Armonk, NY, USA). Wilcoxon-test was used for the comparison of the paired observations. Interclass Correlation Coefficient by Kolmogorov-Smirnov was used to calculate inter- and interobserver variability. P values of < 0.05 were considered statistically significant. The echo templates for Figure three were originally created by Patrick J. Lynch and C. Carl Jaffe, MD and used with permission under the Creative Commons Attribution 2.5 License 2006.

## Results

### Clinical characteristics

Table 1 showed the clinical biological characteristics of SSc patients as well as the results of right heart catheterization. Importantly, all patients had normal mean PAP values.

### Conventional echocardiographic data

Conventional echocardiographic findings are presented in Table 2. There were no differences between baseline and follow-up examination regarding heart rate, left ventricular volumes, aortic valve peak velocity and diastolic function. (Table 2).

**Table 2:** Conventional echocardiographic data

	**Baseline (n = 19)**	**Follow-up (n = 19)**	***p-*** **value**
Heart rate, (bmp)	71.2 ± 11.5	69.7 ± 8.8	ns
LVEDV, (ml)	62.9 ± 21.4	71.8 ± 28.5	ns
LVESV, (ml)	23.5 ± 9.3	27.0 ± 13.7	ns
LV mass index, (g/m^2^)	89.2 ± 14.3	93.8 ± 19.5	ns
Aortic valve peak instantaneous velocity, (m/s)	1.3 ± 0.2	1.4 ± 0.3	ns
***LV diastolic function***			
E, (m/s)	0.70 ± 0.16	0.70 ± 0.15	ns
A, (m/s)	0.69 ± 0.14	0.74 ± 0.14	ns
E/A	1.0 ± 0.3	1.0 ± 0.2	ns
E’, (cm/s)	8.5 ± 2.1	8.4 ± 2.3	ns
A’, (cm/s)	9.3 ± 1.9	9.4 ± 2.1	ns
E/E’	8.4 ± 2.0	8.8 ± 2.8	ns
DT, (ms)	183.1 ± 50.8	176.9 ± 37.6	ns

Data are expressed as mean ± SD. LVEDV, left ventricular enddiastolic volume; LVESV, left ventricular endsystolic volume; LV left ventricular; DT, deceleration time.

### Speckle tracking strain data, DTI systolic velocities and systolic left ventricular function

**Figure 1:**
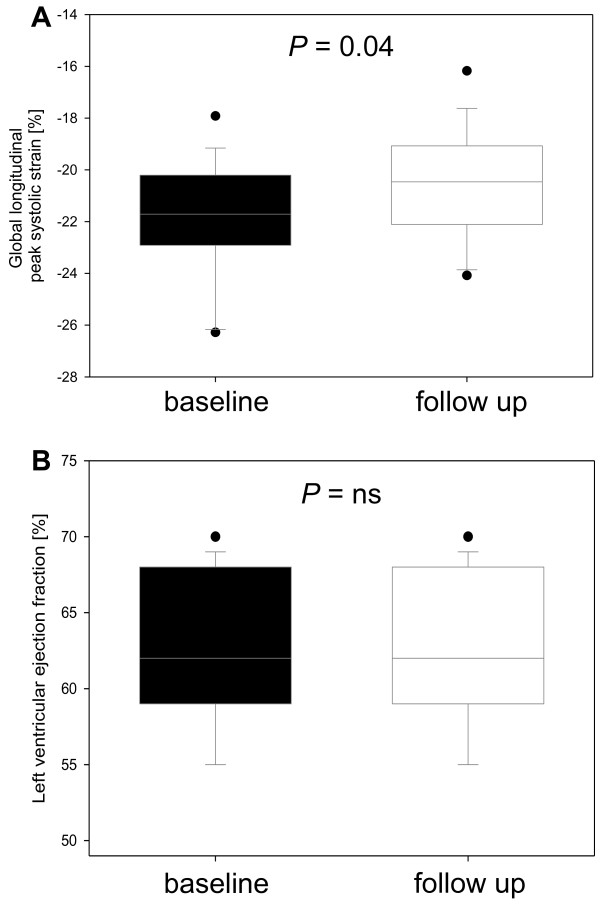
Global longitudinal peak systolic strain (PSS) (A) and left ventricular ejection fraction (B) in systemic sclerosis (SSc) patients at baseline and at follow up.

Table 3 shows global speckle tracking strain data and systolic left ventricular function. LVEF at baseline was normal in all patients and remained stable at follow-up examination (63.3 ± 4.2% vs. 63.2 ± 5.0%, *p* = ns) (Figure 1). In addition, the mean peak systolic velocities were also unchanged (7.9 ± 1.7 vs. 7.9 ± 1.9, *p* = ns).

**Table 3:** Left ventricular ejection fraction, pw DTI and speckle tracking strain data

	**Baseline (n = 19)**	**Follow-up (n = 19)**	***p-*** **value**
LVEF, (%)	63.3 ± 4.2	63.2 ± 5.0	ns
Peak systolic velocity (cm/s)	7.7 ± 1.5	7.8 ± 1.7	ns
***Longitudinal PSS***			
Global longitudinal PSS, (%)	-22.0 ± 2.3	-20.8 ± 2.1	0.04
APLAX, (%)	-21.5 ± 3.5	-20.3 ± 2.7	ns
4CH, (%)	-22.1 ± 2.5	-19.8 ± 3.5	0.006
2CH, (%)	-22.2 ± 2.7	-21.7 ± 2.6	ns

Data are expressed as mean ± SD. Pw DTI, pulsed wave doppler tissue imaging; PSS, peak systolic strain; APLAX, apical long axis view; 4CH, apical four chamber view; 2CH, apical two-chamber view; LVEF, left ventricular ejection fraction.

**Figure 2:**
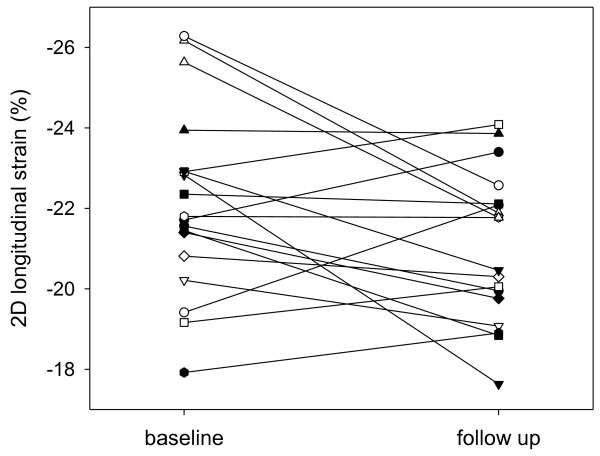
Intraindividual course of the global longitudinal peak systolic strain at baseline (Left Panel) and at follow-up (Right Panel).

The mean global PSS value at follow-up was still in normal range, but significantly reduced compared to baseline examination (baseline -22.0 ± 2.3% vs. follow-up -20.8 ± 3.5%, *p* = 0.04) (Table 3 and Figure 1). Figure 2 shows the intraindividual course of the PSS. This was mainly influenced by lower strain in the four-chamber view (-22.1 ± 2.5% vs. -19.8 ± 3.5%, *p* = 0.006) while there was only a trend for lower strain in the two-chamber view and apical long axis view.

**Figure 3:**
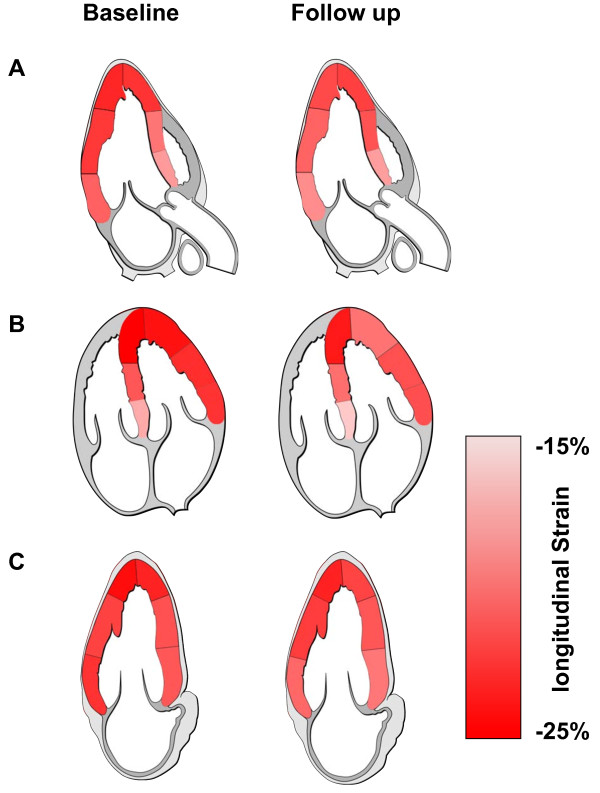
**Colour-coding of the average regional longitudinal peak systolic strain in patients with SSC at baseline (Left Panel) and at follow-up (Right Panel). A**: The apical long axis view. **B**: The four chamber view. **C**: The two chamber view.

Regarding regional analysis we found reduced mean PSS in all segments but only the medial segments in the four-chamber view were statistically significant (Table 4 and Figure 3).

**Table 4:** Regional longitudinal strain data

	**Baseline (n = 19)**	**Follow-up two years (n = 19)**	***p-*** **value**
Basal segments, (%)	-20.3 ± 3.0	-18.8 ± 2.8	0.053
APLAX, (%)	-19.7 ± 4.0	-18.4 ± 3.9	0.286
4CH, (%)	-19.7 ± 3.7	18.2 ± 3.3	0.076
2CH, (%)	-22.4 ± 3.6	-20.2 ± 3.5	0.053
Medial segments, (%)	-21.7 ± 2.3	-21.1 ± 2.6	0.243
APLAX, (%)	-21.4 ± 3.1	-20.9 ± 3.0	0.687
4CH, (%)	-21.9 ± 2.9	-20.6 ± 3.1	0.011
2CH, (%)	-21.4 ± 2.8	-21.8 ± 3.5	0.687
Apical segments, (%)	-23.8 ± 2.9	-22.2 ± 2.6	0.053
APLAX, (%)	-22.6 ± 5.0	-21.6 ± 5.1	0.717
4CH, (%)	-24.5 ± 4.0	-22.4 ± 4.4	0.113
2CH, (%)	-23.7 ± 3.6	-22.9 ± 3.1	0.807

Data are expressed as mean ± SD. PSS, Peak systolic strain; APLAX, apical long axis view; 4CH, apical four-chamber view; 2CH, apical two-chamber view.

### Inter- and intraobserver variability

The intraobserver variability for the LV longitudinal strain was 0.93 (CI 0.79-0.98). The interobserver variability for the LV longitudinal strain was 0.90 (CI 0.57-0.99).

## Discussion

**Figure 4:**
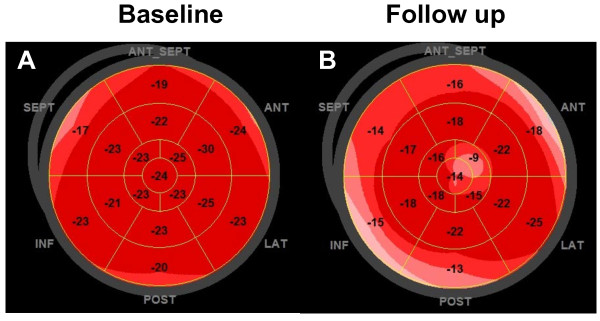
**Bull’s-eye diagram of a patient with SSc at baseline and at follow-up.** Left Panel **(A)**: a global longitudinal peak systolic strain of -21.6% at baseline. Right Panel **(B)**: a global longitudinal peak systolic strain of -16.1% at follow-up (Right Panel, **B**).

Cardiac involvement in patients with SSc is associated with a poor prognosis and is more frequent in SSc patients than clinically suspected [15]. Therefore, a safe, cost-effective, widely available and less observer-dependend diagnostic approach is needed particularly for follow-up visits during the course of the disease. In our study, a minor but significant decrease of the longitudinal LV function was detected by STE in SSc patients with preserved LVEF over a duration of two years (Figure 1, Figure 2, and Figure 4). In contrast, the diastolic function remained stable. Importantly, the peak systolic strain was within the range of normal both at baseline and at follow-up. Accordingly, each examination considered individually would not lead to a therapeutic or diagnostic consequence. Only serial measurements displayed a subclinical decrease in longitudinal function.

Although it has been shown that STE-derived strain is a sensitive marker for the detection of clinical and subclinical myocardial left heart dysfunction in a variety of pathologies, even during follow-up [16], to our knowledge the present study is the first to use STE in patients with SSc as a follow-up tool for daily practice.

In clinical routine, assessment of global systolic function is usually based on the LVEF. This parameter, however, is insufficient in describing the complex myocardial motion. Moreover, LVEF mainly depends on the radial and circumferential deformation caused by less sensitive mid-myocardial and epicardial fibres. In contrast, longitudinal function is predominantly influenced by subendocardial fibres which are most susceptible to evident myocardial damage [17] with preserved LVEF. In addition, despite clear recommendations [13], LVEF is often visually assessed by "eye-balling" with relevant inter- and intraobser variability [18] which inhibits its use for subtle assessment during follow-up. In contrast, 2D speckle tracking derived longitudinal strain is a semiautomatic method that is only minimally affected by inter- and intraobserver variability [9] with with a low test–re-test variability [19] and it can be performed offline from recorded examinations using standard 2D images.

Our regional analysis showed a heterogeneous distribution of declining systolic dysfunction that did not match any particular coronary artery distribution. Histologic studies diagnosed a patchy myocardial fibrosis in up to 80% of patients with SSc [4]. This pattern is well in line with our observation of a dissiminated subclinical decline in regional longitudinal deformation which accordingly may be caused by a slow progression of myocardial fibrosis [3,4]. Due to the disseminated pattern of changes in longitudinal function we emphasize the use of global indices for tracking the systolic function in serial measurements.

No patients had to be excluded due to poor image quality. This indicates an acceptable feasibility of echocardiographic assessment of LV function by speckle tracking in SSc patients.

### Limitations

First, our study was designed as a retrospective, pilot single-center study with only a small number of patients and we cannot draw definite conclusions regarding the prognostic implications of these mild changes in LV function. Additional prospective studies are needed to clarify whether systematic echocardiographic surveillance should be recommended in order to improve the prognosis in this population. Second, we did not track healthy controls over time. Therefore, we cannot completely exclude other variables that might have influenced our results in patients with SSc, e.g. like ageing. Nevertheless, we do not believe in aging effects since it takes decades for longitudinal function to decrease [20]. Third, myocardial fibrosis as a potential reason for a decline in longitudinal function was not assessed by cardiac magnetic resonance imaging (MRI) or myocardial biopsies. Fourth, our study included only long-term scleroderma patients with a median time of 6 years from diagnosis. Therefore, 7 (36.8%) of the included patients had already received potentially cardiotoxic disease-modifying agents (azathioprine, methotrexate or cyclosporine A). Accordingly, we cannot rule out that the reduced longitudinal strain in SSc patients was, at least in part, due to side effects of the medical therapy. Finally, the DETECT study enrolled SSc patients with a reduced diffusing capacity of the lung for carbon monoxide leading to a potential selection bias. Therefore, our results should be transferred with caution to SSc patients without pulmonary involvement.

## Conclusion

STE detect subtle changes of systolic LV function in patients with SSc and preserved LVEF and normal pulmonary pressure after two years. Since cardiac involvement is common, our results may be explained by a progression of subclinical myocardial fibrosis. STE might be a sensititive and valuable method for follow-up of SSc patients, but prospective evaluations are needed for prognostic implications of these changes.

## Competing interests

The authors declare that they have no competing interests.

## Authors’ contributions

SS: Study conception, echocardiographic examinations, data analysis and interpretation, drafting of the manuscript. KR: Acquisition of data and interpretation, participating in statistical analysis. GR: Data analysis, study conception, critical revision of the manuscript. ACB: Data analysis and interpretation, critical revision of the manuscript. SS: Echocardiographic examinations, critical revision of the manuscript. GRB: Data analysis, critical revision of the manuscript. BH: Performed the statistical analysis, data analysis. GB: Study conception and design, data analysis and interpretation. HD: Data analysis and interpretation, preparation of figures, critical revision of the manuscript. FK: Echocardiographic examinations, data analysis and interpretation, revision of the manuscript. All authors read and approved the final manuscript.
